# GlycoRNAs: more than the intersection of glycobiology and RNA biology

**DOI:** 10.1093/lifemedi/lnae044

**Published:** 2024-12-12

**Authors:** Haidong Li, Ruijin Zhang, Runsheng Chen, Jianjun Luo

**Affiliations:** Key Laboratory of Epigenetic Regulation and Intervention, Institute of Biophysics, Chinese Academy of Sciences, Beijing 100101, China; College of Life Sciences, University of Chinese Academy of Sciences, Beijing 100049, China; Key Laboratory of Epigenetic Regulation and Intervention, Institute of Biophysics, Chinese Academy of Sciences, Beijing 100101, China; College of Life Sciences, University of Chinese Academy of Sciences, Beijing 100049, China; Key Laboratory of Epigenetic Regulation and Intervention, Institute of Biophysics, Chinese Academy of Sciences, Beijing 100101, China; College of Life Sciences, University of Chinese Academy of Sciences, Beijing 100049, China; Key Laboratory of Epigenetic Regulation and Intervention, Institute of Biophysics, Chinese Academy of Sciences, Beijing 100101, China; College of Life Sciences, University of Chinese Academy of Sciences, Beijing 100049, China

Rapidly evolving research has accumulated a large amount of evidence showing that messenger RNA (mRNA) and non-coding RNA (ncRNA) contain many different modifications, such as methylation, pseudouridine, hypoxanthine, queuosine, etc. These modifications can affect RNA translation, splicing, and stability, and regulate RNA function. Recently, researchers have discovered a different type of modification, glycosylation, in ncRNA, especially small RNA. This modification is different from other modifications in that a variety of different glycosylation modifications can be produced by changing the length and type of its sugar chain. There is more than one glycosyl group and more than one type of group in the glycosylation modification [1]. These glycosylation modifications are widely present in proteins and are involved in protein localization, folding, and specific functions, but are less studied in RNAs [2].

The term of glycoRNAs (glycosylated RNAs) was coined after the groundbreaking discoveries by Flynn et al. [1], in which they first reported the existence of glycosylated RNA in human cell lines using metabolic chemical reporters. This work is the first to link glycobiology with RNA biology. Follow-up studies by other teams further explored the subcellular localization and function of glycoRNAs, that were found to be located on the cell membrane surface and be able to recruit neutrophils to inflammation sites [3]. All of these suggest that glycoRNAs can utilize glycosyl chains to play important biological roles, and may not just be a simple intersection of glycobiology and RNA biology.

## Discovery of glycoRNAs and modification site

The protein glycosylation process is complex and requires different enzymes to catalyze the synthesis of glycans in different subcellular regions. This process first demands the formation of a core glycan chain, followed by elongation, and branching from the core glycan chain. In humans, up to 15 core glycan chains have been found, which may lead to a wide variety of possible final glycan chains [4]. In RNA modification, the molecular weight of the modification group is very small and may not form a polymer. Although post-translational modifications of proteins are very abundant, glycosylation modification is still the most complex. Due to the excessive focus on RNA sequence, structure, etc., it has never been considered that RNA may be glycosylated. Sialic acid has been reported to be present at the termini of some N-linked glycosylation modifications [4]. In an experiment where Flynn et al. tried to label precursor sugars of glycan, they treated cells with the sialic acid precursor peracetylated *N*-azidoacetylmannosamine (Ac_4_ManNAz) [1]. Ac_4_ManNAz was incorporated into RNA that may undergo N-linked glycosylation during cellular metabolism, while click chemistry enables the conjugation of biotin and cyclooctyne (dibenzocyclooctyne (DBCO)-biotin) to sialic acid. This not only facilitates the use of biotin to visualize glycoRNAs, but also enables the specific enrichment of these glycoRNAs using magnetic beads and RNA sequencing. The analyses indicated that small RNAs such as small nuclear RNA (snRNA), small nucleolar RNAs (snoRNAs), YRNA, transfer RNA (tRNA), ribosomal RNA (rRNA), and 7S RNA were significantly enriched, suggesting that these small RNAs were likely to be glycosylated. In addition, another study developed a different method to enrich glycosylated RNAs, and found that miRNAs were also enriched [5].

However, these studies still could not directly prove the existence of glycoRNAs. Until recently, Xie et al. used mass spectrometry technology to provide more direct evidence for the existence of glycoRNA [6]. They utilized RNase A and PNGase F to digest and isotopically label the enriched glycoRNAs, and found the modification site for glycoRNAs at the nucleotide level, namely acp^3^U (3-(3-amino-3-carboxypropyl)uridine). Previous studies have reported that acp^3^U is present in tRNA [7]. Therefore, they speculated that tRNA is likely to be glycosylated at the acp^3^U site. The DTW domain containing (DTWD) family is the enzyme that catalyzes the formation of acp^3^U on tRNA. When DTWD was knocked out, the glycosylation level decreased slightly, suggesting that acp^3^U is likely to serve as a glycosylation site to form glycosylation modification on tRNA, but the glycosylation site is not limited to acp^3^U.

## Transport and localization of glycoRNAs

**Figure 1.**
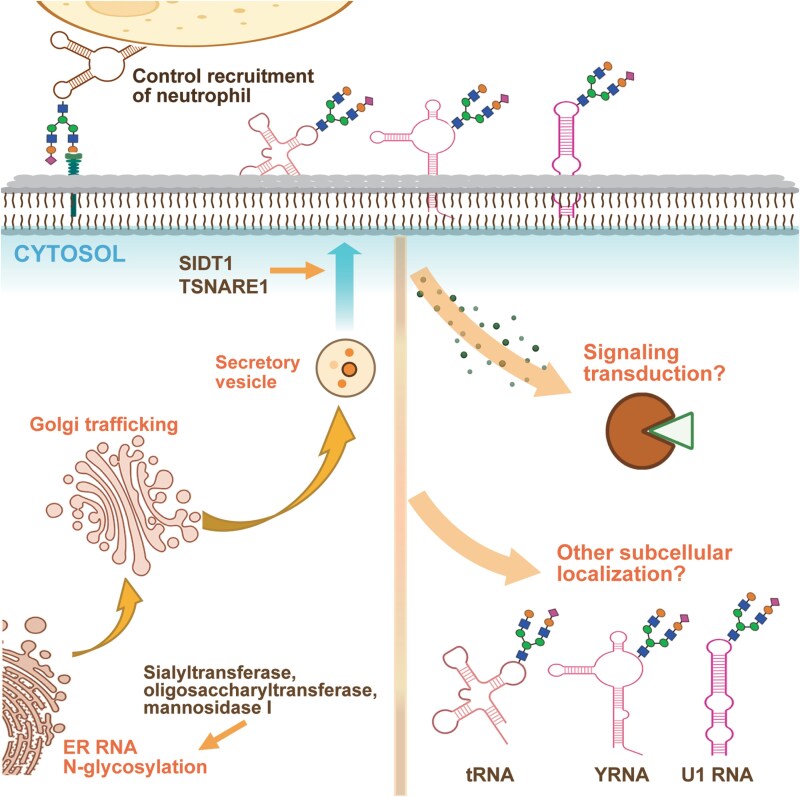
**Overview of glycosylation and the transport and properties of glycoRNAs.** Emerging evidence suggests that glycoRNAs, like glycosylated membrane proteins, undergo glycosylation in the endoplasmic reticulum and Golgi apparatus, and are subsequently transported to the cell membrane via vesicular trafficking. Inhibition of the three glycosylases results in reduced levels of glycoRNA, and co-localization imaging showed that glycoRNA co-localized with TSNARE and VTl1B, two proteins responsible for vesicle docking and fusion at the cell membrane. In addition, glycosylated RNA can be localized in the lipid raft region of the cell membrane, and the channel protein SIDT1 that mediates transmembrane RNA transport also plays a role in the localization of glycosylated RNA to the cell membrane. Functionally, glycoRNAs have been identified on the surface of neutrophils, where they may assist in targeting neutrophils to sites of inflammation. Research on glycoRNAs is still in its early stages, particularly regarding their functional roles. For instance, given that glycoRNAs can localize to the cell membrane and carry glycosylation modifications, could they act as membrane receptors involved in signal transduction? Additionally, could glycoRNAs localize to other subcellular regions to perform specific biological functions? These questions remain to be explored.

In order to properly perform their functions, biological macromolecules need to be localized to a specific location. The study by Flynn et al. [1] used a lectin-based proximity labeling method to discover RNAs located near the cell membrane; however, proximity labeling could only show that these RNAs were located near glycans. The modified proximity ligation technology developed by Ma et al. was composed of two single-stranded DNAs, one of which contains an aptamer that could bind to a glycan, and the other is complementary to target RNA [8]. Using this technology, it was found that three types of glycoRNAs may be transported to the cell membrane via the vesicle pathway and co-localized with lipid raft regions. In the study for glycoRNA recruitment of neutrophils, it was found that SID1 transmembrane family member 1 (SIDT1) plays a key role in the localization of glycoRNAs on the outer cell membrane [3]. The transport and localization of biological macromolecules is a dynamic process involving interactions between various biological macromolecules and cell components, which is very complex. Although there have been initial explorations of the transportation and localization of glycoRNAs, there are still many key questions that remain unanswered. For example, are there sequences similar to signal peptides in proteins that determine the localization of glycoRNAs? Which molecules aid in the transportation of glycoRNAs to their destination? What is the specific molecular mechanism? Where else can glycoRNAs be localized in the subcellular region? These questions still require a large number of experiments to clarify (Fig. 1).

## Functions of glycoRNAs and their relevance to human health

Modifications on RNA require specific proteins, known as readers, to recognize these modifications in order to exert their functions. Most RNA modifications discovered so far are known to function intracellularly. In contrast, glycosylation modifications found in proteins and lipids primarily act extracellularly, contributing to processes such as immune evasion, regulation of cell-to-cell interactions, and participation in cell signaling [2]. Could glycoRNAs similarly exert specific functions through readers in the extracellular environment? Research has for the first time shown that glycoRNAs recognized by P-selectin on the cell surface can function to help endothelial cells recruit neutrophils to inflammation sites to participate in immune responses [3]. When glycoRNAs on the cell surface are digested with RNase and PNGase, endothelial cells can no longer recruit neutrophils. Moreover, during the differentiation process of macrophages, the content of glycoRNAs changes significantly. Studies have shown that when RNA modifications become dysregulated, they may contribute to the development of cancer [9]. GlycoRNAs show heterogeneous expression in different malignant metastatic cancer cell lines, suggesting that these glycoRNAs may be involved in the process of cancer occurrence. Although it has been demonstrated that glycoRNAs can participate in immune responses and are heterogeneously expressed in cancer cell lines, it is still unclear which glycoRNAs are involved in immune responses and how they affect cancer initiation. Given the complexity of glycosylation modifications, there are certainly many biological functions involving glycoRNAs that remain to be discovered.

GlycoRNAs are like the only plant in the desert. When we discover it and try to uproot it, we would marvel at how deeply and intricately its roots are planted. The RNA world hypothesis posits that in the early stages of life, RNA both served as a storage for genetic information and had biological functions. In the process of evolution, the function of storing genetic information was replaced by the more stable DNA, and the biological function was taken over by proteins, with RNA serving as an intermediary between proteins and DNA. The term “RNA world” was first proposed by Walter Gilbert in 1868 [10]. And scientists have found many pieces of evidence, such as RNA acting as an enzyme catalyzing the synthesis of peptide bonds, and being reverse-transcribed into DNA. Therefore, glycoRNAs are likely remnants of the RNA world, filling in the gaps but leaving much to be explored further.
